# Automatic Prediction of MGMT Status in Glioblastoma via Deep Learning-Based MR Image Analysis

**DOI:** 10.1155/2020/9258649

**Published:** 2020-09-23

**Authors:** Xin Chen, Min Zeng, Yichen Tong, Tianjing Zhang, Yan Fu, Haixia Li, Zhongping Zhang, Zixuan Cheng, Xiangdong Xu, Ruimeng Yang, Zaiyi Liu, Xinhua Wei, Xinqing Jiang

**Affiliations:** ^1^Department of Radiology, Guangzhou First People's Hospital, Guangzhou Medical University, Guangzhou 510180, China; ^2^Sun Yat-sen University, Guangzhou, China; ^3^Philips Healthcare, Guangzhou, China; ^4^EPFL, Lausanne, Switzerland; ^5^Department of Radiology, Guangdong Provincial People's Hospital, Guangdong Academy of Medical Sciences, Guangzhou 510080, China

## Abstract

Methylation of the O^6^-methylguanine methyltransferase (MGMT) gene promoter is correlated with the effectiveness of the current standard of care in glioblastoma patients. In this study, a deep learning pipeline is designed for automatic prediction of MGMT status in 87 glioblastoma patients with contrast-enhanced T1W images and 66 with fluid-attenuated inversion recovery(FLAIR) images. The end-to-end pipeline completes both tumor segmentation and status classification. The better tumor segmentation performance comes from FLAIR images (Dice score, 0.897 ± 0.007) compared to contrast-enhanced T1WI (Dice score, 0.828 ± 0.108), and the better status prediction is also from the FLAIR images (accuracy, 0.827 ± 0.056; recall, 0.852 ± 0.080; precision, 0.821 ± 0.022; and *F*_1_ score, 0.836 ± 0.072). This proposed pipeline not only saves the time in tumor annotation and avoids interrater variability in glioma segmentation but also achieves good prediction of MGMT methylation status. It would help find molecular biomarkers from routine medical images and further facilitate treatment planning.

## 1. Introduction

Glioblastoma multiforme (GBM) is the most common and aggressive type of primary brain tumor in adults. It accounts for 45% of primary central nervous system tumors, and the 5-year survival rate is around 5.1% [1, 2]. The standard treatment for GBM is surgical resection followed by radiation therapy and temozolomide (TMZ) chemotherapy, which improves median survival by 3 months compared to radiotherapy alone [3]. Several studies indicated that O^6^-methylguanine-DNA methyltransferase (MGMT) gene promoter methylation reported in 30-60% of glioblastomas [4] can enhance the response to TMZ, which has been proven to be a prognostic biomarker in GBM patients [3, 5]. Thus, determination of MGMT promoter methylation status is important to medical decision-making.

Genetic analysis based on surgical specimens is the reference standard to assess the MGMT methylation status, while a large tissue sample is required for testing MGMT methylation status using methylation-specific polymerase chain reaction [6]. In particular, the major limitations are the possibility of incomplete biopsy samples due to tumor spatial heterogeneity and high cost [7]. Besides, it cannot be used for real-time monitoring of the methylation ﻿status.

Magnetic resonance imaging (MRI) is a standard conventional examination in diagnosis, preoperative planning, and therapy evaluation of GBM [8, 9]. Recently, radiomics, extracting massive quantitative features from medical images, has been proposed to explore the correlation between image features and underlying genetic traits [10–12]. There is growing evidence that radiomics can be used in predicting the status of MGMT promoter methylation [13–15]. However, most previous works utilized handcrafted features. This procedure includes tumor segmentation, feature extraction, and informatics analysis [16–19]. In particular, tumor segmentation is a challenging and important step because most works depend on manual delineation. This step is burdensome and time consuming, and inter- or intraobserver disagreement is unavoidable. Deep learning which can extract features automatically has been emerging as an innovative technology in many fields [20]. The convolutional neural network (CNN) is proven to be effective in image segmentation, disease diagnosis, and other medical image analysis tasks [21–25]. Compared to traditional methods with handcrafted features, deep learning shows several advantages of being robust to distortions such as changes in shape and lower computational cost. A few studies have shown that deep learning can be used to segment tumors and predict MGMT methylation status for glioma [26]. However, to the best of our knowledge, there is no previous report regarding building a pipeline for both glioma tumor segmentation and MGMT methylation status prediction in an end-to-end manner. Therefore, we investigate the feasibility of integrating the tumor segmentation and status prediction of GBM patients into a deep learning pipeline in this study.

## 2. Methods

### 2.1. Data Collection

A total of 106 GBM patients were analyzed in our study. MR images, including presurgical axial contrast-enhanced T1-weighted images (CE-T1WI) and T2-weighted fluid-attenuated inversion recovery (FLAIR) images, were collected from The Cancer Imaging Archive (http://www.cancerimagingarchive.net). The images were originated from four centers (Henry Ford Hospital, University of California San Francisco, Anderson Cancer Center, and Emory University). Clinical and molecular data were also obtained from the open-access data tier of the TCGA website.

Genomic data were from the TCGA data portal. MGMT methylation status analysis was performed on Illumina HumanMethylation27 and HumanMethylation450 BeadChip platforms. A median cutoff using the level 3 beta-value present in the TCGA was utilized for categorizing methylation status. Illumina Human Methylation probes (cg12434587 and cg12981137) were selected in this study [27].

Of 106 GBM cases, 87 cases were with CE-T1W images, and 66 cases with FLAIR images. We randomly split the cases into training and testing sets with the ratio of 8 : 2 and applied 10-fold cross-validation to the training set with scikit-learn library (https://scikit-learn.org/stable/). The dataset distribution is listed in Table 1.

### 2.2. Image Preprocessing

**Figure 1:**
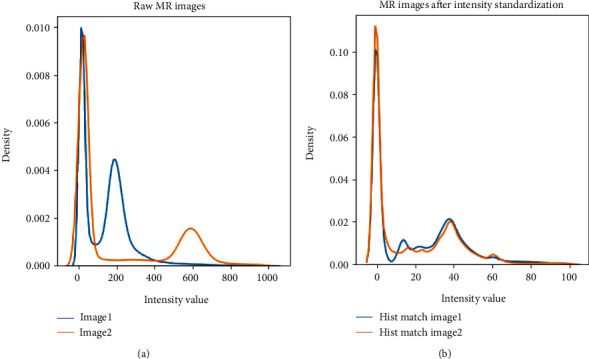
Density plot of two different MR images (a) before and (b) after piece-wise linear histogram matching.

For general images, the pixel values contain reliable image information. However, MR images do not have a standard intensity scale. In Figure 1(a), we show the density plot of two raw MR images. In each plot, there are two peaks, the peak around 0 refers to background pixels, and the other peak refers to white matter. The white matter peaks of the two images are far away. Thus, MR images normalization is needed to guarantee that the grey values of the same tissue among different MR images are close to each other [28].

The piece-wise linear histogram matching was used to normalize the intensity distribution of MR images [29]. Firstly, we studied standard histogram distribution via averaging the 1st to 99th percentile of all images. Then, we linearly mapped the intensities of each image to this standard histogram. In Figure 1(b), we can see that the white matter peaks of two images coincide with each other after normalization. Secondly, the images were normalized to zero mean and unit standard deviation only on valued voxels. At last, data augmentation was used to increase the dataset size to avoid overfitting. We rotated images for every 5 degrees from -20 to +20 degrees, resulting in a 9-fold increment in the number of MRI scans.

### 2.3. Segmentation

**Figure 2:**
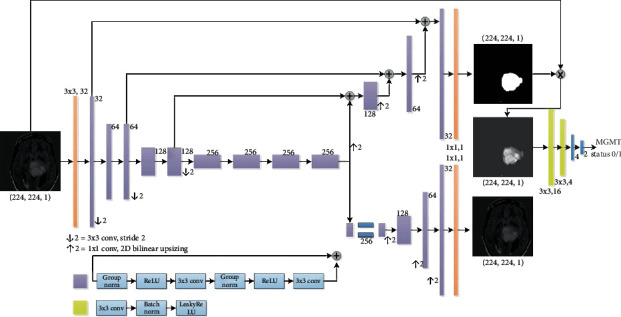
An end-to-end deep learning pipeline for both tumor segmentation and status classification.

As for tumor segmentation, one state-of-the-art model [30] in BraTS 2018 challenge (Multimodal Brain Tumor Segmentation 2018 Challenge http://braintumorsegmentation.org/) was adapted. The whole network architecture is shown in Figure 2.

In short, the deep learning model added a variational autoencoder (VAE) branch to a fully convolutional network model. The decoder part was shared for both segmentation and VAE tasks. The prior distribution taken for the KL divergence in the VAE part is *N*(0, 1). ResNet blocks used in the architecture [31] included two 3 × 3 convolutions with normalization and ReLU as well as skip connections. In the encoder part, the image dimension was downsampled using stride convolution by 2 and increased channel size by 2. For the decoder part, the structure was similar to that of the encoder part but using upsampled. The decoder endpoint had the same size as the input image followed by sigmoid activation, and its output was for tumor segmentation. As for the VAE part, the encoder output was reduced to 256, and the input image was reconstructed by using a similar structure as the decoder without skip connection. The segmentation part output the tumor segmentation and the VAE branch attempted to reconstruct the input image. Except for the input and output layers, all blocks in Figure 2 utilized the ResNet block with different channel numbers (depicted aside each layer). For the input layer, a 3 × 3 convolution was with 3 channels; and for both output layers, a 3 × 3 convolution with a dropout rate of 0.2 and *L*_2_ regularization with weight 1*e* − 3 were used to avoid overfitting. The loss function consists of 3 terms as shown in

(1) $$\begin{array}{c} L=L_{\text{Dice}}+0.1\times L_{L2}+0.1\times L_{KL}, \end{array}$$

where *L*_Dice_ is the soft Dice loss between the predicted segmentation and the ground truth labels. The ground truth labels were manually annotated with ImageJ (https://imagej.nih.gov) by one neuroradiologist with 10 years' experience specialized in brain disease diagnosis. *L*_*L*2_ is the *L*_2_ loss on the VAE branch output image and the input image, and *L*_*KL*_ is the standard VAE penalty term [32, 33]. Then, the Dice coefficient as defined in function (2) was calculated to assess the performance of segmentation:

(2) $$\begin{array}{c} \text{Dice}=\underset{i}{\sum }\frac{2\cdot p_{i}\cdot \hat{p_{i}}}{{\left\|p_{i}\right\|}^{2}+{\left\|\overset{\wedge }{p_{i}}\right\|}^{2}+\text{epsilon}}, \end{array}$$

where *p*_*i*_ is the ground truth, ${\hat{p}}_{i}$ is the prediction for pixel *i*, and epsilon = 1*e* − 8.

### 2.4. Status Classification

Meanwhile, for the classification of MGMT methylation status, a 4-layer CNN was designed. Further, the classification model was cascaded with the tumor segmentation model. At the stage of the tumor segmentation model design, the classification network was tried with different numbers of convolutional layers [2–5], and we found that 2 convolutional layers with 2 fully connected (FC) layers performed the best for this task. The first convolutional layer had 16 filters, and the second one had 4 filters. All the convolutional layers had a kernel size of 3 × 3 and stride of 1 followed by LeakyReLU, batch normalization, and max pooling. LeakyReLU was an advanced ReLU activation that avoids dead neurons by setting a negative half-axis slope 0.3 instead of 0. Its advantages include good performance in eliminating gradient saturation, low computational cost, and faster convergence. Batch normalization was used to normalize features by the mean and variance within a small batch. It helped to solve the covariance shift issue and ease optimization. Max pooling with a 4 × 4 filter was used to downsample image features extracted through convolutional layers and then fed into 2 FC layers. ReLU and softmax were adapted as activation functions for the first and second FC layers, respectively. The weight initialization of all layers was done by He-normal [34].

### 2.5. Parameter Settings and Software

All experiments were conducted under the open-source framework Keras (https://keras.io/) on one GeForce RTX 2080Ti GPU. The numbers of parameters of the segmentation and classification model are, respectively, 6,014,721 and 3,498. In tumor segmentation, Adam optimizer was adapted with a self-designed learning rate scheduler which was initialized with a learning rate 1*e* − 4; then, the learning rate was divided by 2 when the validation loss did not reduce in the past 5 epochs. The epoch was set at 50 and batch size at 8. Every epoch took around 50 seconds. In tumor classification, 4-CNN was trained for 50 epochs which utilized Adam with learning rate 2*e* − 4, and the batch size was 32. If the validation accuracy was observed stable for over 10 epochs, the training process would be ended. The averaged elapsed time for each epoch was 5 seconds.

### 2.6. Statistical Analysis

The Dice coefficient was calculated for evaluating the performance of tumor segmentation. For the MGMT methylation status classification, the accuracy rate, recall, precision, and *F*_1_ score were calculated according to equations listed below. In addition, the receiver operating characteristic (ROC) curve was plotted, and the area under the ROC curve (AUC) was reported to measure the classification accuracy. All the parameters were calculated in PyCharm with the programming language of Python (version 3.6.8; Wilmington, DE, USA; http://www.python.org/):

(3) $$\begin{array}{c} \text{Accuracy}=\frac{\text{TP}+\text{TN}}{\text{TP}+\text{TN}+\text{FP}+\text{FN}},\\ \text{Recall}=\frac{\text{TP}}{\text{TP}+\text{FN}},\\ \text{Precision}=\frac{\text{TP}}{\text{TP}+\text{FP}},\\ F_{1}=\frac{2\times \text{Precision}\times \text{Recall}}{\text{Precision}+\text{Recall}}, \end{array}$$

where TP is the true positive, TN is the true negative, FP is the false positive, and FN is the false negative.

## 3. Results

### 3.1. Tumor Segmentation

#### 3.1.1. Qualitative Observation

**Figure 3:**
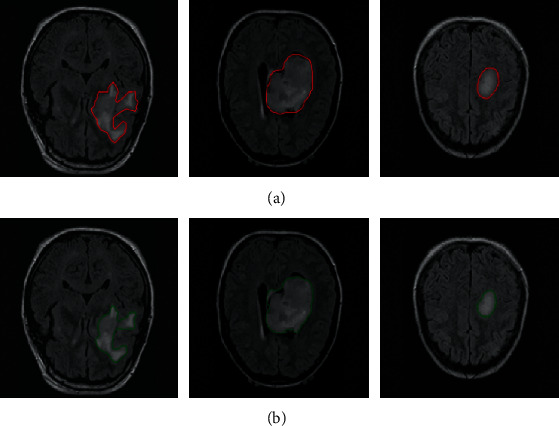
Automatic segmentation results of brain tumors with FLAIR images. (a) The ground truth of tumor boundaries in FLAIR images and (b) automatic segmentation results using the proposed network with FLAIR images.

Tumors could be accurately delineated by the proposed pipeline.  Figure 3 shows the annotated ground truth (the first row) and corresponding segmentation results (the second row) of GBM in FLAIR images. It is observed that tumor boundaries could be accurately localized by using the deep learning network, and the major hyperintense regions are delineated. The three cases show that automatic segmentation is quite close to the ground truth.

**Figure 4:**
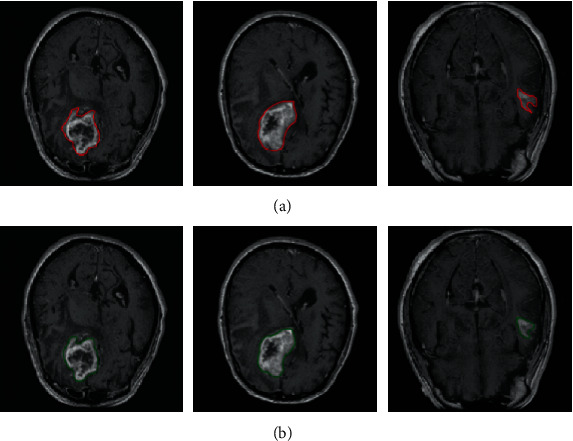
Three representative cases of brain tumor manual annotation and automatic segmentation with CE-T1WI images. (a) The manual annotation and (b) the automatic segmentation results with our proposed network.

Figure 4 shows the GBM in CE-T1WI images, and the ground truth (the first row) and the segmentation results (the second row) are presented. Tumor boundaries are localized, and it seems that there is no obvious difference between the manual annotation and its corresponding segmentation results obtained from our proposed network, and the suspicious regions are mainly contoured. The three cases show that segmentation results from the deep network approximate the manual delineation.

#### 3.1.2. Quantitative Evaluation

The quantitative performance of automatic tumor segmentation is summarized in Table 2. The deep network obtained good testing performance on tumor segmentation using CE-T1WI (Dice score, 0.828 ± 0.108) and FLAIR (Dice score, 0.897 ± 0.007). And the Dice scores from FLAIR were slightly higher than those from CE-T1WI across training, validation, and testing sets. The maximum difference of the Dice score between average Dice scores from CE-T1W images in training and validation sets was 0.026, indicating that the model was not overfitting.

#### 3.1.3. Computational Performance

Time consumption between manual annotation and automatic prediction per MR slice is compared as shown in Table 3. For the evaluation of time consumption, we recorded the total time and divided it by the number of slices. So, the time listed in Table 3 was the average segmentation time per slice. It was observed that the deep network was more efficient, and it took less than 0.2 seconds to complete the segmentation of an MR slice, while manual annotation required more than 30 seconds.

### 3.2. Classification of MGMT Promoter Methylation Status

Table 4 shows the prediction performance of MGMT promoter methylation status which is evaluated from four classification metrics (accuracy, recall, precision, and *F*_1_ score) on three stages (training, validation, and testing) when using different MR images (CE-T1WI, FLAIR). In general, the model trained with FLAIR achieves better results for all metrics across three stages, followed by the model trained with CE-T1WI images. Specifically, the accuracy, recall, precision, and *F*_1_ score of the deep model trained with FLAIR images reach 0.827, 0.852, 0.821, and 0.836 in the testing stage, respectively.

**Figure 5:**
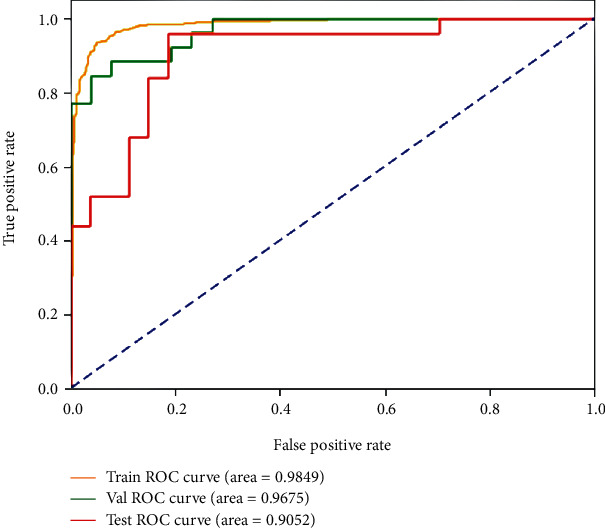
ROC curves of the best result on the FLAIR images for MGMT promoter methylation status classification on the training, validation, and testing datasets.

**Figure 6:**
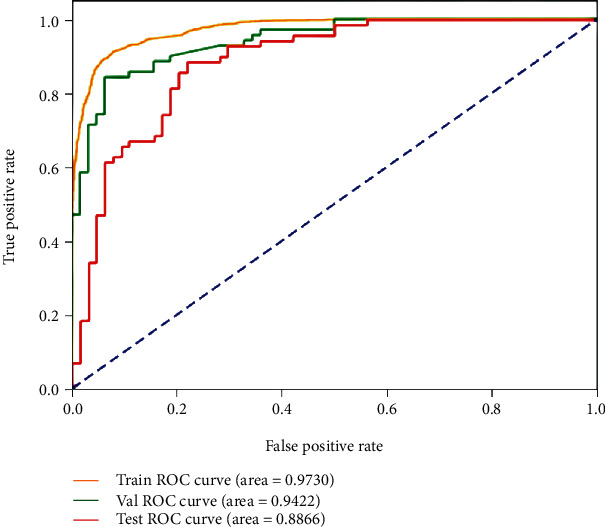
ROC curves of the best result on the CE-T1W images for MGMT promoter methylation status classification in the training, validation, and testing datasets.

ROC curves of the prediction results are demonstrated in Figures 5 and 6.  Figure 5 shows the best status classification when using FLAIR images for a deep model, which achieves an AUC of 0.985 (yellow curve), 0.968 (green curve), and 0.905 (red curve) on the training, validation, and testing datasets, respectively.

The best status classification when using CE-T1WI images for deep model training is shown in Figure 6. The well-trained deep model obtains AUC up to 0.973 (yellow curve), 0.942 (green curve), and 0.887 (red curve) on the training, validation, and testing datasets, respectively.

## 4. Discussion

This study presents an MR-based deep learning pipeline for automatic tumor segmentation and MGMT methylation status classification in an end-to-end manner for GBM patients. Experimental results demonstrate promising performance on accurate glioma delineation (Dice score, 0.897) and MGMT status prediction (accuracy, 0.827; recall, 0.852; precision, 0.821; and *F*_1_ score, 0.836) coming from the model trained with FLAIR images. In addition, the proposed pipeline dramatically shortens the inference time on glioma segmentation.

For glioma segmentation, one state-of-the-art deep model is utilized and obtains impressive performance on the involved MGMT dataset for GBM segmentation. Its performance is close to these deep network-based tumor segmentation studies. Hussain et al. [35] reported a CNN approach for glioma MRI segmentation, and the model achieved a Dice score of 0.87 on the BRATS 2013 and 2015 datasets. Cui et al. [36] proposed an automatic semantic segmentation model on the BRATS 2013 dataset, and the Dice score was near 0.80 on the combined high- and low-grade glioma datasets. Kaldera et al. [37] proposed a faster RCNN method and achieved a Dice score of 0.91 on 233 patients' data. These studies suggest that deep networks are full of potential for accurate tumor segmentation in MR images.

Several deep models have been designed for the classification of MGMT methylation status in GBM patients. Chang et al. [38] proposed a deep neural network which achieved a classification accuracy of 83% for 259 gliomas patients with T1W, T2W, and FLAIR images. Korfiatis et al. [26] compared different sizes of the ResNet baseline model and reached the highest accuracy of 94.9% in 155 GBM patients with T2W images. Han et al. [39] proposed a bidirectional convolutional recurrent neural network architecture for MGMT methylation classification, while the accuracy was around 62% for 262 GBM patients with T1W, T2W, and FLAIR images. In this study, a shallow CNN is used, and the classification performance is promising. The best performance comes from the model trained with FLAIR images, and we achieved a satisfactory result with the highest accuracy of 0.827 and recall of 0.852 in consideration of the relatively small dataset.

In the previous studies, Drabycz et al. [40] analyzed handcrafted features to distinguish methylated from unmethylated GBM and figured out that texture features from T2-weighted images were important for the prediction of MGMT methylation status. Han et al. [41] found that MGMT promoter-methylated GBM was prone to more tumor necrosis, while T2-weighted FLAIR sequence may be more sensitive to necrosis than T1-weighted images. Interestingly, we also find that better performances of both GBM segmentation and molecular classification are achieved on FLAIR images in our study although the images of CE-T1W and FLAIR did not come from the same patients.

The strengths of this study lie in the fully automatic glioma segmentation and predicting the MGMT methylation status based on a small dataset. Generally, it takes a radiologist about one minute per slice in tumor annotation, while the inference time of the deep learning model is about 0.1 seconds which is around 1/600 times used in manual annotation. Additionally, manual annotation is burdensome and prone to introduce inter- and intraobserver variability. While once well trained, a deep learning model can continuously and repeatedly perform tumor segmentation regardless of the observers. On the other hand, the training strategy in this study is beneficial for small dataset analysis. In general, a deep model requires a large number of training instances. However, it is challenging or impossible to provide massive high-quality images in medical imaging. Finally, although several studies tried to use deep networks for automatic glioma segmentation [35, 36, 42] or molecular classification [26, 38, 39], the proposed network in this study could integrate both glioma segmentation and classification in a seamless connection pipeline. And the performance is competitive to the state-of-the-art studies in tumor segmentation and classification.

There are several limitations to our study. First, the sample size is small in the study; we will further confirm the findings in a study with larger samples. Second, a multicenter research trial is helpful to validate the capability of the proposed pipeline, while the variations of MR imaging sequences, equipment venders, and other factors could impose difficulties on model building. Third, we failed to investigate the value of combined CE-T1WI and FLAIR in tumor segmentation and classification considering the fewer samples. In the future, we will explore multiple MR sequences for MGMT methylation status prediction, such as amide-proton-transfer-weighted imaging and diffusion-weighted imaging. These may have great potential to improve the performance of MGMT methylation status prediction.

## 5. Conclusion

An MRI-based end-to-end deep learning pipeline is designed for tumor segmentation and MGMT methylation status prediction in GBM patients. It can save time and avoid interobserver variability in tumor segmentation and help discover molecular biomarkers from routine medical images to aid in diagnosis and treatment decision-making.

## Figures and Tables

**Table 1:** Dataset distribution of each experiment.

	Phase	Cases (methylation/unmethylation)	CE-T1WI slices (methylation/unmethylation)	FLAIR slices (methylation/unmethylation)
FLAIR	Training	51 (25/26)	676 (288/388)	—
	Testing	15 (7/8)	167 (62/105)	
CE-T1WI	Training	70 (36/34)	—	1208 (609/599)
	Testing	17 (10/7)		220 (109/111)

Note: FLAIR: fluid-attenuated inversion recovery; CE-T1WI; contrast-enhanced T1-weighted imaging.

**Table 2:** Dice scores of the deep network on tumor segmentation using MR images.

Modality	Training	Validation	Testing
CE-T1WI	0.832 ± 0.009	0.831 ± 0.012	0.828 ± 0.108
FLAIR	0.893 ± 0.004	0.892 ± 0.008	0.897 ± 0.007

Note: the number in the table referred to the mean ± standard deviation values of 10 cross-validation experiments. CE-T1WI: contrast-enhanced T1-weighted imaging; FLAIR: fluid-attenuated inversion recovery.

**Table 3:** Inference time (seconds) of one MR slice for glioma segmentation.

Modality	Manual annotation	Deep model
CE-T1WI	50 s	0.11 s
FLAIR	60 s	0.07 s

Note: CE-T1WI: contrast-enhanced T1-weighted imaging; FLAIR: fluid-attenuated inversion recovery.

**Table 4:** Results of MGMT methylation status classification.

Modality	Phase	Classification			
		Accuracy	Recall	Precision	*F*_1_ score
CE-T1WI	Training	0.894 ± 0.012	0.906 ± 0.007	0.886 ± 0.018	0.896 ± 0.010
	Validation	0.839 ± 0.046	0.866 ± 0.044	0.823 ± 0.051	0.845 ± 0.045
	Testing	0.804 ± 0.011	0.818 ± 0.033	0.798 ± 0.014	0.808 ± 0.015

FLAIR	Training	0.941 ± 0.056	0.943 ± 0.104	0.947 ± 0.026	0.945 ± 0.081
	Validation	0.885 ± 0.090	0.941 ± 0.105	0.857 ± 0.028	0.889 ± 0.101
	Testing	0.827 ± 0.056	0.852 ± 0.080	0.821 ± 0.022	0.836 ± 0.072

Note: the number in the table referred to the mean ± standard deviation values of 10 cross-validation experiments. CE-T1WI: contrast-enhanced T1-weighted imaging; FLAIR: fluid-attenuated inversion recovery.

## Data Availability

All MRI data are available in the cancer imaging archive (https://www.cancerimagingarchive.net/), and clinical and molecular data are obtained from the open-access data tier of the TCGA website.
